# Edge States, Bulk Spectra, and Topological Phases of Szegedy’s Quantum Search on a One-Dimensional Cycle with Self-Loops

**DOI:** 10.3390/e27060623

**Published:** 2025-06-12

**Authors:** Mengke Xu, Xi Li, Xunan Wang, Wanglei Mi, Xiao Chen

**Affiliations:** 1 School of Information Engineering, China Jiliang University, Hangzhou 310018, China; xmk22@cjlu.edu.cn (M.X.); xunan@cjlu.edu.cn (X.W.); 22h034160135@cjlu.edu.cn (W.M.); 2 School of Software, Henan University, Zhengzhou 450046, China; lixi_nuli@yeah.net

**Keywords:** topological phases, edge states, three-fold degenerate points, quantum search, Chern number

## Abstract

Topological transitions are relevant for boundary conditions. Therefore, we investigate the bulk spectra, edge states, and topological phases of Szegedy’s quantum search on a one-dimensional (1D) cycle with self-loops, where the search operator can be formulated as an open boundary condition. By establishing an equivalence with coined quantum walks (QWs), we analytically derive and numerically illustrate the quasienergies dispersion relations of bulk spectra and edge states for Szegedy’s quantum search. Interestingly, novel gapless three-band structures are observed, featuring a flat band and three-fold degenerate points. We identify the topological phases ±2 as the Chern number. This invariant is computed by leveraging chiral symmetry in zero diagonal Hermitian Hamiltonians that satisfy our quasienergies constraints. Furthermore, we demonstrate that the edge states enhance searches on the marked vertices, while the nontrivial bulk spectra facilitate ballistic spread for Szegedy’s quantum search. Crucially, we find that gapless topological phases arise from three-fold degenerate points and are protected by chiral symmetry, distinguishing ill-defined topological transition boundaries.

## 1. Introduction

Discrete-time quantum walks (QWs) display rich features, outperforming their classical counterparts, such as quantum speed-up in walk [1,2], localization in the two-dimensional (2D) Grover walk [3], and the topological transitions in a split-step QW [4,5]. To realize searches, different coins act on marked vertices [6,7]. Inspired by coin operators, Szegedy proposed a quantum search algorithm giving rise to a quadratic speed-up [8]. Szegedy’s quantum search can also achieve a speed-up on the one-dimensional (1D) cycle with self-loops, and localization can occur on marked vertices, as described in our previous work [9]. Since bound states manifest as localization at different topological phase boundaries, we focus on the topological phases of Szegedy’s quantum search on the 1D cycle with self-loops.

Interest in topological phases has grown significantly in recent years. While naturally occurring topological insulators remain rare, QWs can be engineered to simulate dynamics in a wide range of topological phases, such as split-step QWs [4,10], QWs with different coins at boundaries [11,12,13,14], and non-Hermitian QWs [15,16]. The nontrivial topological properties are manifested by robust edge states at the phase boundaries (i.e., the zero energy bound states in 1D or the gapless edge modes in 2D) [17,18,19,20]. Remarkably, owing to topologically protected edge states, an effective transport channel from one site to another site is ensured in disordered 2D QWs by cutting some links [21]. This observation naturally raises two key questions: (1) can edge states emerge in quantum search algorithms? (2) How might they influence Szegedy’s quantum search framework, given that the search operator effectively mimics open boundary conditions?

Furthermore, Szegedy’s QW on the 1D cycle with self-loops is equivalent to two iterations of three states’ QWs (3QWs) with self-loops [22]. The 3QWs have a three-band structure, which exhibits outstanding features in the presence of three-fold degenerate points [18]. Intriguingly, low energy excitations in the three-band model can be accurately described by pseudospin-1 Maxwell equations in Hamiltonian form and three-fold degenerate points called Maxwell points [23]. Subsequently, an experiment of topological Maxwell metal bands was conducted in a superconducting qutrit [24]. Given these findings, a key question arises: how do three-fold degenerate points influence the topological properties of Szegedy’s QW?

We have demonstrated that Szegedy’s quantum search on a 1D cycle with self-loops exhibits localization at the marked vertices [9]. The observation that 1D edge states demonstrate localization has inspired our topological investigation for quantum searches on such self-loop cycle graphs in [18,19]. Szegedy’s search operator is viewed as an open boundary condition, which leads to an edge state along the marked vertices. Building upon the equivalence between Szegedy’s quantum walk and two steps of the generalized three-state quantum walk, we analyze the quasienergies and dispersion relations of both bulk spectra and edge states. A three-band structure is observed, consisting of a flatband [25] and three-fold degenerate points. Then, to calculate the Chern number as topological phases of bulk spectra and edge states, it is expected to have a Hamiltonian. However, we do not use the given known Hamiltonians from physical models but try to find the Hamiltonians caused by the known quasienergies. Consequently, for a Hermitian Hamiltonian with zero diagonal elements, possessing chiral symmetry and satisfying the quasienergies conditions, the bulk spectra and edge states all have nontrivial topological phases (the Chern number). The topological phases originate from the three-fold degenerate points and are protected by certain symmetry. Furthermore, we should emphasize that nontrivial edge states can enhance transport. In contrast, the nontrivial bulk spectra imply the ballistic spread for Szegedy’s QW on the 1D cycle with self-loops.

The paper is structured as follows. Section 2 begins with a review of Szegedy’s quantum walk and search framework. In Section 3, the quasienergies of bulk spectra and edge states of the generalized three-state quantum search are obtained. Subsequently, the quasienergies dispersion relations of Szegedy’s quantum search are derived in Section 4. Proceeded by finding the unknown Hamiltonians induced by the known quasienergies, topological phases depended on the diagonal and certain symmetry are analyzed in Section 5. Conclusions are sketched in Section 6.

## 2. Preliminaries

**Figure 1:**
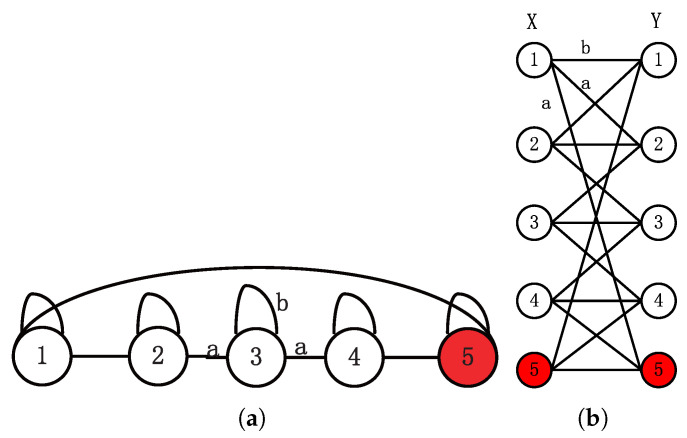
(**a**) The 1D cycle with self-loops *b* of n=5,m=1. (**b**) Bipartite graph of Szegedy’s QW.

In this Section, we begin by introducing Szegedy’s quantum search on the 1D cycle with self-loops [8]. As depicted in Figure 1a, n=5,m=1, *n* is the number of vertices, *m* is the number of marked vertices denoted by red, the outdegree *a* is equal to the indegree, and *b* is the weight of self-loops. To develop a general quantum Markov chain, Szegedy devised two reflection operators derived from a bipartite graph by duplicating vertices sets, as pictured in Figure 1b.

Let *X* and *Y* be two finite vertices sets from the bipartite graph and $P_{x,y}$, $Q_{y,x}$ be probability transition matrices denoted probability from *X* to *Y* and *Y* to *X*. By the definition of probability transition matrices, we have $\sum_{y}p_{x,y}=1,\;\forall x\in X,\;\sum_{x}q_{y,x}=1,\;\forall y\in Y$. If X=Y, we set P=Q.

To quantize the walk, the two operators $A:H^{n}\to H^{n^{2}}$ and $B:H^{n}\to H^{n^{2}}$ are obtained on the Hilbert space $H^{n^{2}}=H^{n}⊗H^{n}$ with basis states $\left\{|x\rangle \left.|y\rangle \left|x\in X,y\in Y\right.\right\}\right.$ as follows:

(1) $$\begin{array}{c} A=\sum_{x\in X}|\alpha_{x}\rangle \langle x|,\;\;\;\;\;\;\;\;\;\;\;\;\;\;\;\;\;\;\;\;B=\sum_{y\in Y}|\beta_{y}\rangle \langle y|, \end{array}$$

where $|\alpha_{x}\rangle =\sum_{y\in Y}\sqrt{p_{x,y}}|x\rangle |y\rangle$, and $\;|\beta_{y}\rangle =\sum_{x\in X}\sqrt{q_{y,x}}|x\rangle |y\rangle$.

Furthermore, according to the constructions of matrices *A* and *B*, we can derive the following:

(2) $$\begin{array}{c} A^{T}A=I_{n\times n},\;\;\;\;B^{T}B=I_{n\times n},{\left(AA^{T}\right)}^{2}=AA^{T},{\left(BB^{T}\right)}^{2}=\;BB^{T}, \end{array}$$

where the characters of $AA^{T}$ and $BB^{T}$ are akin to the projector operators.

Thereby, two reflection operators, $R_{A}$ and $R_{B}$, are defined by the following:

(3) $$\begin{array}{c} R_{A}=2AA^{T}-I_{n^{2}\times n^{2}},R_{B}=2BB^{T}-I_{n^{2}\times n^{2}}. \end{array}$$

Thus, the QW operator is denoted by the product of two reflection operators:

(4) $$\begin{array}{c} U=R_{B}R_{A}. \end{array}$$

To search a marked vertex, the modified stochastic matrix $P^{\prime }$ is given as follows:

(5) $$\begin{array}{c} p_{x,y}^{\prime }=\left\{\begin{array}{c} p_{x,y},\;\;\;\;\;\;\;\;\;\;\;x\notin M;\\ \delta_{x,y},\;\;\;\;\;\;\;\;\;\;\;x\in M, \end{array}\right.\; \end{array}$$

where *M* is a marked vertex set.

Furthermore, the quantum search operator transforms into $U^{\prime }=R_{B}^{\prime }R_{A}^{\prime }$.

**Figure 2:**
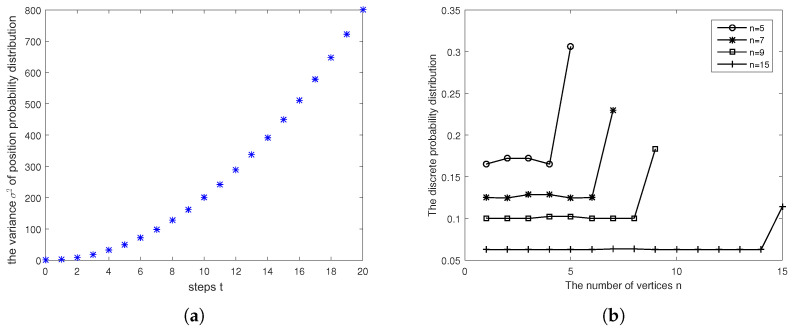
(**a**) The variance of the position probability distribution for Szegedy’s quantum search on the 1D cycle with self-loops (parameters: b=0.8,n=5,m=1) exhibits ballistic transport over steps evolution *t*. (**b**) The discrete probability distribution of Szegedy’s quantum search (parameters: b=0.8,m=1) for the number of vertices n=5,7,9,15 after a long-time evolutionary process. The marked vertex is localized at the last position. The success probability of finding the marked vertex remains large.

In our previous study of Szegedy’s quantum search on the 1D cycle with self-loops, we demonstrated that Szegedy’s quantum search is ballistic spreading on marked vertices, as shown in Figure 2a. In addition, we found that the probability of finding a marked vertex remains high after the long-time evolutionary process, as depicted in Figure 2b, indicating the presence of edge states (localized bound states) at the marked vertices boundary. For further details, we refer reader to [9].

Motivated by the existence of these localized bound states, we concentrate on the edge states of Szegedy’s quantum search on the 1D cycle with self-loops.

## 3. Bulk Spectra and Edge States of the Generalized Three States Quantum Search

Prior to examining the bulk spectra and edge states of Szegedy’s quantum search on the 1D cycle with self-loops, we first consider the generalized 3QWs since Szegedy’s QW on the 1D cycle with self-loops is identical to two iterators of 3QWs. First, we discuss the quasienergies of the generalized 3QWs. Subsequently, the modified search operator enables us to observe edge states.

### 3.1. Bulk Spectra of the Generalized 3QWs

The 3QW is governed by three degrees of freedom $\left\{|L\rangle ,|O\rangle ,|R\rangle \right\}$, representing left-moving, stationary, and right-moving states, respectively. In its standard form, the walk is defined by transition probability $a=\frac{1}{3}$ for turning left or right and probability $b=\frac{1}{3}$ for staying at the same location. Notably, the 3QW is the simplest model to exhibit localization [26]. In this paper, we take the case of a 3QW ruled by three degrees of freedom with any probability satisfied 2a+b=1 (*a* is the turning left or right probability and *b* is the staying at the same location probability). This case is called a generalized 3QW here, which is also the 1D cycle with self-loops in a finite system.

In general, the quantum evolution of the generalized 3QWs is produced through successive applications of a QW operator:

(6) $$\begin{array}{c} W=S_{0}\left(C⊗I_{3\times 3}\right), \end{array}$$

where *C* denotes the coin operator,

(7) $$\begin{array}{c} C=2|s_{c}\rangle \langle s_{c}|-I_{3\times 3}=\left[\begin{array}{ccc} 2a-1 & 2\sqrt{ab} & 2a\\ 2\sqrt{ab} & 2b-1 & 2\sqrt{ab}\\ 2a & 2\sqrt{ab} & 2a-1 \end{array}\right], \end{array}$$

with superposition state in the coin space $|s_{c}\rangle ={\left[\begin{array}{ccc} \sqrt{a} & \sqrt{b} & \sqrt{a} \end{array}\right]}^{T}$, $\left(2a+b=1,a,b\in R^{+}\right)$, where $S_{0}$ denotes the conditional shift operator as follows:

(8) $$\begin{array}{c} S_{0}=\sum_{l\in Z}\left\{|l-1\rangle \langle l|⊗|L\rangle \langle L|+|l\rangle \langle l|⊗|O\rangle \langle O|+|l+1\rangle \langle l|⊗|R\rangle \langle R|\right\}, \end{array}$$

which coherently couples the position space $\left(l\in Z\right)$ to the three-state coin space $\left\{|L\rangle ,|O\rangle ,|R\rangle \right\}$.

In a set of Fourier transformations $|k\rangle =\frac{1}{\sqrt{N}}\sum_{l}e^{-ikl}|l\rangle$, −π≤k≤π, the walk operator *W* in Equation (6) can be written as follows:

(9) $$W\left(k\right)=S_{0}\left(k\right)C=\left[\begin{array}{ccc} e^{ik} & 0 & 0\\ 0 & 1 & 0\\ 0 & 0 & e^{-ik} \end{array}\right]\left[\begin{array}{ccc} 2a-1 & 2\sqrt{ab} & 2a\\ 2\sqrt{ab} & 2b-1 & 2\sqrt{ab}\\ 2a & 2\sqrt{ab} & 2a-1 \end{array}\right],$$

where $W\left(k\right)$ refers to quantities with a dependence on *k* in this paper.

The explicit eigenvalue expressions of the walk operator $W\left(k\right)$ in Equation (9) are calculated as follows:

(10) $$\begin{array}{c} \lambda_{1}=1,\;\;\;\;\lambda_{2,3}=e^{\pm iE},\;\;\;\;\;\;\;\cos\left(E\right)=-2a-b\cos k. \end{array}$$

On the other hand, a QW can be regarded as a stroboscopic simulator of an effective Hamiltonian H(k) as follows:

(11) $$\begin{array}{c} W\left(k\right)=e^{-iH(k)}. \end{array}$$

Based on Equation (11) above, the quasienergies of the Hamiltonian H(k) associated with the walk operator $W\left(k\right)$ are obtained:

(12) $$\begin{array}{c} E_{1}=0,E_{2,3}=\pm arc\cos\left(-2a-b\cos k\right), \end{array}$$

where k=0 and $E_{2}=-E_{3}=\pi$.

In particular, the quasienergy $E_{1}=0$ is a flat band in the middle of the three bands.

### 3.2. Edge Spectra of the Generalized Three-State Quantum Search

We establish a search algorithm based on the generalized 3QWs. To realize search, the walk operator *W* is modified to the search operator $W^{\prime }=S_{0}C^{\prime }$,

(13) $$\begin{array}{c} W^{\prime }=S_{0}\left\{\begin{array}{c} \left(C⊗I_{3\times 3}\right),\;\;\;\;\;\;\;\;\;\;\;on\;\;\;\;unmarked\\ -I_{3\times 3},\;\;\;\;\;\;\;\;\;\;\;\;\;\;\;\;\;\;\;\;\;\;\;\;\;\;\;\;\;\;\;\;\;\;\;\;\;on\;\;\;\;\;\;\;marked \end{array},\right. \end{array}$$

where the matrix $I_{3\times 3}$ denotes the identity matrix. The search operator is constructed by modifying the coin operator on marked vertices. Throughout this work, primed notation (′) distinguishes search-related operators from their original counterparts in QWs.

Substituting Equation (13) into Equation (9) to yield the quantum search operator $W^{\prime }=S_{0}C^{\prime }$ on marked vertices in Fourier space, the following is realised:

(14) $$W^{\prime }\left(k\right)=S_{0}\left(k\right)C^{\prime }=\left[\begin{array}{ccc} e^{ik} & 0 & 0\\ 0 & 1 & 0\\ 0 & 0 & e^{-ik} \end{array}\right]\left[\begin{array}{ccc} -1 & 0 & 0\\ 0 & 1 & 0\\ 0 & 0 & -1 \end{array}\right].$$

Similarly, the eigenvalues of the search operator $W^{\prime }\left(k\right)$ are calculated as follows:

(15) $$\begin{array}{c} \lambda_{1}^{\prime }=1,\;\;\;\;\lambda_{2,3}^{\prime }=-e^{\pm iE^{\prime }},\cos\left(E^{\prime }\right)=-\cos k. \end{array}$$

Subsequently, the quasienergies of the effective Hamiltonian $H^{\prime }\left(k\right)$ are obtained using the analytical formulas $W^{\prime }\left(k\right)=e^{-iH^{\prime }\left(k\right)}$,

(16) $$\begin{array}{c} E_{1}^{\prime }=0,E_{2,3}^{\prime }=\pm \left(\pi -arc\cos\left(\cos k\right)\right). \end{array}$$

When k=±π, $E_{1}^{\prime }=E_{2}^{\prime }=E_{3}^{\prime }=0$.

Fundamentally, according to [27], we denote BS as follows:

(17) $$\begin{array}{c} BS=E_{1}\cup \left(E_{2},\pi -E_{2}\right)\cup \left(-\pi +E_{2},-E_{2}\right), \end{array}$$

as the quasienergies dispersion relations of bulk spectra, where $E_{1},E_{2}$ is in Equation (12).

Similarly, we denote ES as follows:

(18) $$\begin{array}{c} ES=E_{1}^{\prime }\cup \pm \left(\pi -\arccos\left(\cos k\right)\right)\cup \pm \arccos\left(\cos k\right) \end{array}$$

as the quasienergies dispersion relations of edge states in our systems, where $E_{1}^{\prime }$ is in Equation (16) and −π≤k≤π.

**Figure 3:**
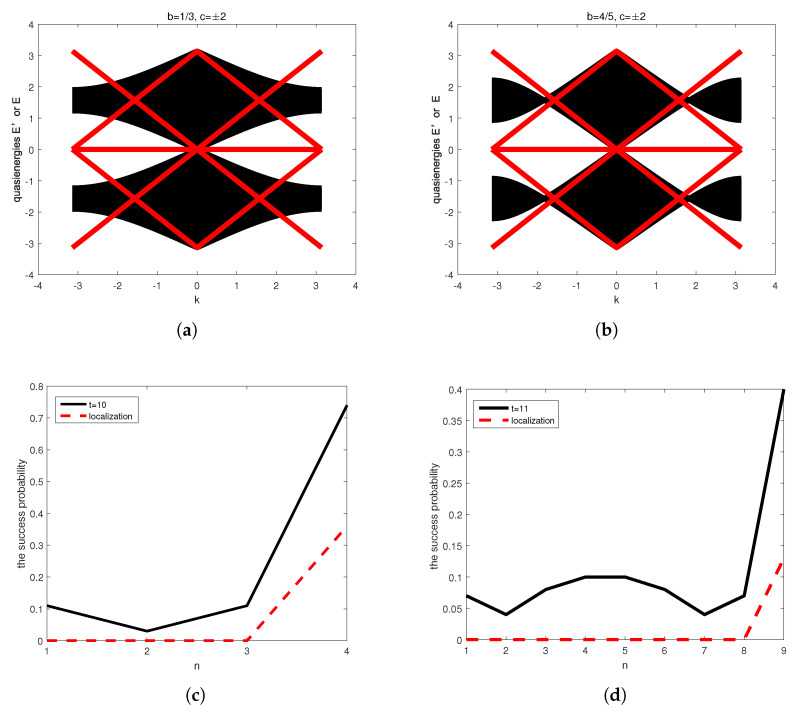
(**a**,**b**): The quasienergy dispersion relations of bulk spectra denoted by the black regions in Equation (17) and edge states denoted by the red lines in Equation (18) for the generalized 3QWs. (**a**) With self-loops, $b=\frac{1}{3}$; (**b**) with self-loops, $b=\frac{4}{5}$. (**c**,**d**) The success probability of reaching each vertex after *t* steps of Szegedy’s search on a 1D cycle with self-loops (black solid line) and the localized probability after long-time evolution (red dashed line). (**c**) for n=5, m=2, t=10, and $b=\frac{4}{5}$ (five vertices, two of which are marked vertices after 10 steps); (**d**) for n=10, m=2, t=11, and $b=\frac{1}{3}$. The last two vertices are marked and treated equivalently as a single vertex. The topological phase is denoted by *c*.

Figure 3a,b illustrate the quasienergies dispersion relations for systems with weighted self-loops $b=\frac{1}{3}$ and $b=\frac{4}{5}$, respectively. The quasienergies structures are three bands constituted by a flat band and three-fold degenerate points. The dispersions of bulk spectra depicted by the black regions have the three-fold degenerate points at (0,0), while edge states depicted by the red lines show additional degeneracies at (0,0) and $\left(\pm \pi ,0\right)$. Thereby, they are gapless. Obviously, the edge states traversing these bands can be clearly seen. In fact, the flat band of bulk spectra corresponds to localization at the origin, arising from the degenerate eigenvalue 1 for the generalized 3QWs. The parameters c=±2 stand for topological phases of lower and higher quasienergies bands, as will be discussed in detail in Section 5.

In one-dimensional topological systems, the manifestation of edge states in quantum walks is characterized by localized probability distributions at the boundaries. This is demonstrated in Figure 3c,d, which presents the success probability of Szegedy’s quantum search with self-loops (black solid line, calculated in [28]) after *t* steps of evolution, as well as the long-time localized probability at marked vertices (red dashed line, calculated in [9]). In Figure 3c, the parameters are n=5, m=2, t=10, and $b=\frac{4}{5}$ (five vertices, two of which are marked vertices after 10 steps). Clearly, localization occurs at the marked vertices four and five. In Figure 3d, the parameters are n=10, m=2, t=11, and $b=\frac{1}{3}$. Here, localization emerges at the marked vertices 9 and 10, further demonstrating that edge states in quantum searches exhibit spatial localization at marked vertices. Note that the last two vertices are treated as a single marked vertex in this analysis.

## 4. Bulk Spectra and Edge States of Szegedy’s Quantum Search on the 1D Cycle with Self-Loops

We now turn our attention back to Szegedy’s quantum search on the 1D cycle with self-loops. We obtain the quasienergies of bulk spectra by the equivalence between Szegedy’s QW on the 1D cycle with self-loops and the two iterations of the generalized 3QWs. In terms of searches, we express the search operator as an open boundary condition, which leads to edge states. Furthermore, we also discuss the generalized 3QWs with different coin operators as iterations.

### 4.1. Bulk Spectra of Szegedy’s QW

The connection between Szegedy’s QW and the generalized 3QWs allows us to formulate Szegedy’s walk operator $U=R_{B}R_{A}$ in Equation (4) explicitly:

(19) $$\begin{array}{c} U=R_{B}R_{A}=SR_{A}SR_{A}=WW, \end{array}$$

where $W=S_{0}\left(C⊗I_{3\times 3}\right)$ denotes the walk operator of the generalized 3QWs in Equation (6).

Thereby, Szegedy’s walk operator $U=R_{B}R_{A}$ in Fourier space is rewritten by the following:

(20) $$\begin{array}{c} U(k)=W(k)W(k), \end{array}$$

where $W\left(k\right)$ is Equation (9).

The eigenvalues of Szegedy’s walk operator U(k) are calculated as follows:

(21) $$\begin{array}{c} \begin{array}{c} \lambda_{1}=1,\;\lambda_{2,3}=e^{\pm iE},\cos\left(E\right)=2b^{2}\cos^{2}k+8ab\cos k+8a^{2}-1. \end{array} \end{array}$$

Similarly, based on $U\left(k\right)=e^{-iH(k)}$ and −π≤k≤π, the quasienergies of the corresponding Hamiltonian H(k) are yielded:

(22) $$\begin{array}{c} \begin{array}{c} E_{1}=0,E_{2,3}=\pm arc\cos\left(2b^{2}\cos^{2}k+8ab\cos k+8a^{2}-1\right). \end{array} \end{array}$$

When k=0, $E_{1}=E_{2}=E_{3}=0$.

### 4.2. Edge Spectra of Szegedy’s Quantum Search

With respect to quantum searches, for an unmarked vertex x∈X, Szegedy’s search reflection operator $R_{A}^{\prime }=2A^{\prime }A^{\prime T}-I_{n^{2}\times n^{2}}$ from Equation (5) applies to the unmarked vertex *x*,

(23) $$\begin{array}{c} \begin{array}{c} {R^{\prime }}_{A}c_{x,y}|x,y\rangle =R_{A}c_{x,y}|x,y\rangle =\left(2\sum_{\left|x-w\right|\leq 1}\sqrt{p_{x,y}p_{x,w}}c_{x,w}-c_{x,y}\right)|x,y\rangle , \end{array} \end{array}$$

whose effect on an unmarked vertex is identical to the walk reflection operator $R_{A}=2AA^{T}-I_{n^{2}\times n^{2}}$ in Equation (3). The $c_{x,y}$ denotes the amplitude of state $|x,y\rangle$. *y* and *w* denote vertices connected to the unmarked vertex *x* in the graph, where $p_{x,y}$ represents elements of the probability transition matrix $P_{x,y}$.

For a marked m∈M, where *M* is a set of marked vertices, however, the marked state $|\alpha_{m}\rangle$ transforms to $|\alpha_{m}\rangle =|m,m\rangle$ since $p_{m,m}^{\prime }=1$ in Equation (5). Then, the reflection operator $R_{A}^{\prime }$ acts on an edge joining m∈M and y∈Y by the following:

(24) R′Am,y=2m,mm,mm,mm,ym,y−m,y=m,m,y=m;−m,y,y≠m.

In short, the search reflection operator $R_{A}^{\prime }$ has opposite signs to the marked vertices (except the self-loops) and inverts the average amplitude of the unmarked vertices around the subspace of the joint vertices. The second reflection operator $R_{B}^{\prime }=2B^{\prime }B^{\prime T}-I_{n^{2}\times n^{2}}$ in the set of *Y* is the same as $R_{A}^{\prime }$ due to $R_{B}^{\prime }=SR_{A}^{\prime }S$, where $S|i,j\rangle =|j,i\rangle$.

Due to reflective effects, the search reflection operator $R_{A}^{\prime }$ acting on the marked vertices can be regarded as an open boundary condition along the marked vertices. To illustrate this explicitly, we use the example of $n=5,m=1,b=\frac{1}{3}$ shown in Figure 1b to show how we deal with the marked vertices. The last vertex is a marked vertex depicted by the red circle. Our analysis particularly examines the evolution of state $|4,5\rangle$, as the amplitude of the unmarked vertices adjacent to the marked vertices increases the probability of finding a marked vertex.

The first reflection operator $R_{A}^{\prime }$ inverts the average amplitude of unmarked vertex 4, then the amplitude $c_{4,5}$ of state $|4,5\rangle$ changes to $c_{4,5}^{\prime }$. Subsequently, the second reflection operator $R_{B}^{\prime }=SR_{A}^{\prime }S$ acting on the set of *Y* flips the signs of the incident edges for the marked vertex 5∈Y, while preserving the self-loop state $|5,5\rangle$. Hence, the evolution of the amplitude in one step of Szegedy’s quantum search for the state $|4,5\rangle$ is shown as follows:

(25) $$\begin{array}{c} c_{4,5}|4,5\rangle \overset{{R^{\prime }}_{A}}{\to }c_{4,5}^{\prime }|4,5\rangle \overset{S}{\to }c_{4,5}^{\prime }|5,4\rangle \overset{{R^{\prime }}_{A}}{\to }-c_{4,5}^{\prime }|5,4\rangle \overset{S}{\to }-c_{4,5}^{\prime }|4,5\rangle . \end{array}$$

The previous analysis allows us to obtain the expression of Szegedy’s search operator $U^{\prime }=R_{B}^{\prime }R_{A}^{\prime }$ from Equation (5) on the marked vertices in the Fourier space under the open boundary condition:

(26) $$\begin{array}{c} U^{\prime }\left(k\right)=S_{0}\left(k\right)C^{\prime }S_{0}\left(k\right)C\\ =\left[\begin{array}{ccc} e^{ik} & 0 & 0\\ 0 & 1 & 0\\ 0 & 0 & e^{-ik} \end{array}\right]\left[\begin{array}{ccc} -1 & 0 & 0\\ 0 & 1 & 0\\ 0 & 0 & -1 \end{array}\right]\left[\begin{array}{ccc} e^{ik} & 0 & 0\\ 0 & 1 & 0\\ 0 & 0 & e^{-ik} \end{array}\right]\left[\begin{array}{ccc} 2a-1 & 2\sqrt{ab} & 2a\\ 2\sqrt{ab} & 2b-1 & 2\sqrt{ab}\\ 2a & 2\sqrt{ab} & 2a-1 \end{array}.\right] \end{array}$$

In the analogy, the eigenvalues of Szegedy’s search operator $U^{\prime }\left(k\right)$ can be obtained as follows:

(27) $$\begin{array}{c} \begin{array}{c} {\lambda^{\prime }}_{1}=1,\;\;{\lambda^{\prime }}_{2,3}=-e^{\pm iE^{\prime }},\cos\left(E^{\prime }\right)=\cos\left(-2a+b\cos2k\right). \end{array} \end{array}$$

The quasienergies of the Hamiltonian $H^{\prime }\left(k\right)$ from $U^{\prime }\left(k\right)=e^{-iH^{\prime }\left(k\right)}$ are provided as follows:

(28) $$\begin{array}{c} E_{1}^{\prime }=0,E_{2,3}^{\prime }=\pm arc\cos\left(-2a+b\cos2k\right). \end{array}$$

When $k=\pm \frac{\pi }{2}$, $E_{2}^{\prime }=-E_{3}^{\prime }=\pi$.

**Figure 4:**
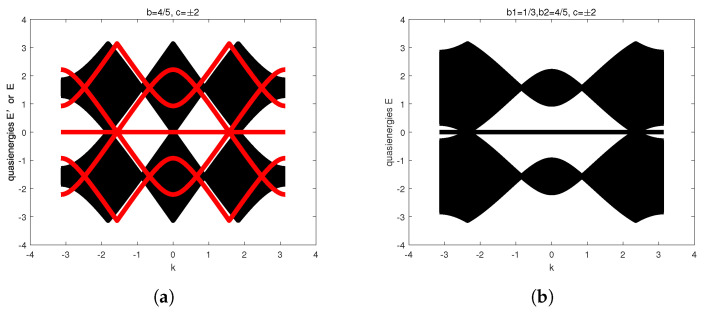
(**a**) The quasienergy dispersion relations of bulk spectra denoted by the black regions in Equation (22) and edge states denoted by the red lines in Equation (28) for Szegedy’s quantum search on the 1D cycle with self-loops $b=\frac{4}{5}$. (**b**) The quasienergies dispersion relations of bulk spectra denoted by the black regions in Equation (33) for the generalized 3QWs with the first coin operator of the self-loops $b_{1}=\frac{1}{3}$ and the second coin of $b_{2}=\frac{4}{5}$. The topological phase is denoted by *c*.

Using Equations (17) and (18), the quasienergies dispersion relations of bulk spectra and edge states for Szegedy’s quantum search with weighted self-loops $b=\frac{4}{5}$ are illustrated in Figure 4a. The black regions denote the bulk spectra, whereas the red line denotes the edge states. The three-band structures are gapless, regardless of bulk spectra or edge states. The flat band corresponds to localization at the origin. However, the three-fold degenerate points of bulk spectra are different from edge states.

### 4.3. Bulk Spectra of the Generalized 3QWs with Different Coin Operators as Iterations

Given the equivalence between Szegedy’s QW operator and two iterations of the generalized 3QWs with identical coin operators, we further investigate the case of the generalized 3QWs with different coin operators at two iterations.

Based on Equation (6), the walk operator $V=W_{2}W_{1}$ for the generalized 3QWs with different coin operators at the two iterations is given by the following:

(29) $$\begin{array}{c} V=W_{2}W_{1}=S_{0}\left(C_{2}⊗I_{3\times 3}\right)S_{0}\left(C_{1}⊗I_{3\times 3}\right), \end{array}$$

where $C_{1}$ and $C_{2}$ are different coin operators with different weights of self-loops $b_{1},b_{2}$, and $S_{0}$ is the conditional shift operator.

Putting it in the Fourier space, Equation (29) transforms into the following:

(30) $$\begin{array}{c} V\left(k\right)=S_{0}\left(k\right)C_{2}S_{0}\left(k\right)C_{1}, \end{array}$$

where

(31) $$\begin{array}{c} C_{2}=\left[\begin{array}{ccc} 2a_{2}-1 & 2\sqrt{a_{2}b_{2}} & 2a_{2}\\ 2\sqrt{a_{2}b_{2}} & 2b_{2}-1 & 2\sqrt{a_{2}b_{2}}\\ 2a_{2} & 2\sqrt{a_{2}b_{2}} & 2a_{2}-1 \end{array}\right]; \end{array}$$

(32) $$\begin{array}{c} C_{1}=\left[\begin{array}{ccc} 2a_{1}-1 & 2\sqrt{a_{1}b_{1}} & 2a_{1}\\ 2\sqrt{a_{1}b_{1}} & 2b_{1}-1 & 2\sqrt{a_{1}b_{1}}\\ 2a_{1} & 2\sqrt{a_{1}b_{1}} & 2a_{1}-1 \end{array}\right]. \end{array}$$

Similarly, the quasienergies of Hamiltonian H(k) corresponding to the walk operator $V=W_{2}W_{1}$ are,

(33) $$\begin{array}{c} \begin{array}{c} E_{1}=0,\\ E_{2}=arc\cos\left(\begin{array}{c} \left(4a_{1}a_{2}-2a_{1}-2a_{2}+1\right)\cos2k-2a_{2}\\ -2a_{1}+12a_{1}a_{2}+\;8\sqrt{a_{1}a_{2}b_{1}b_{2}}\cos k \end{array}\right),\\ E_{3}=-E_{2}. \end{array} \end{array}$$

When $b_{1}=b_{2},k=0$, $E_{1}=E_{2}=E_{3}=0$.

Similar to Equations (17) and (18), Figure 4b depicts the quasienergy dispersion relations of bulk spectra for the generalized 3QWs with the first coin operator of the self-loops $b=\frac{1}{3}$ and the second coin operator of $b=\frac{4}{5}$. Compared to Figure 4a, the three-fold degenerate points have been changed. The parameters c=±2 characterize topological phases associated with the lower and higher quasienergy bands, as quantified by their Chern numbers. A detailed discussion of these topological properties follows in the next section.

## 5. Topological Phases

Having established the quasienergy dispersion relations for bulk spectra and edge states of Szegedy’s quantum search on the 1D cycle with self-loops, we now focus on characterizing their topological phases through Chern number calculations. Hence, we need to determine a Hamiltonian H(k) in the Fourier space to calculate the Chern number of the three eigenstates. Unlike the given Hamiltonians from physical models, we establish a Hamiltonian operator H(k) in Fourier space that satisfies the quasienergies obtained in Section 3 and Section 4. Thus, we address the following question: how do we determine whether a correct Hamiltonian H(k) satisfied the following quasienergies: $E_{1}=0$, $E_{3}=-E_{2}$, and the given $E_{2}\in R$ ? $E_{2}$ is from Equations (12), (16), (22), and (28).

A Hamiltonian H(k) can support topological phases if it possesses a specific symmetry. The bulk-boundary correspondence of chiral symmetric QWs was explored [29,30]. In our system, the quasienergies have $E_{2}=-E_{3}$ and Szegedy’s QW has a bipartite structure; therefore, chiral symmetry (CS) is considered in this paper. A operator possesses CS: if $\left\{|\psi^{A}\rangle ,|\psi^{B}\rangle \right\}$ is an eigenstate of eigenvalue *E*, then $\left\{\pm |\psi^{A}\rangle ,\mp |\psi^{B}\rangle \right\}$ is an eigenstate of eigenvalue −E [31].

Given that the Hamiltonian H(k) for a generalized 3QW takes the form of a 3×3 matrix, we begin by postulating the following general Hermitian operator structure:

(34) $$\begin{array}{c} H\left(k\right)=\left[\begin{array}{ccc} h_{11} & h_{12} & h_{13}\\ h_{12}^{*} & h_{22} & h_{23}\\ h_{13}^{*} & h_{23}^{*} & h_{33} \end{array}\right], \end{array}$$

where $tr\left(H(k)\right)=0$, and $\left|H(k)\right|=0$, requiring the following:

(35) $$\begin{array}{c} \left\{\begin{array}{c} h_{11}h_{33}+h_{22}h_{33}+h_{11}h_{22}-h_{12}h_{12}^{*}-h_{13}h_{13}^{*}-h_{23}h_{23}^{*}=-E_{2}^{2},\\ -h_{11}h_{22}h_{33}-h_{12}h_{23}h_{13}^{*}-h_{13}h_{12}^{*}h_{23}^{*}+h_{22}h_{13}h_{13}^{*}+h_{33}h_{12}h_{12}^{*}+h_{11}h_{23}h_{23}^{*}=0. \end{array}\right. \end{array}$$

Note that all matrix elements $h_{i,j}$ are *k*-dependent quantities (i.e., $h_{i,j}\left(k\right)$ for i,j∈{1,2,3} with−π≤k≤π), but we left out *k* to simplify the notation. The symbol (*) denotes the complex conjugation.

The analysis of the topological phases depends on the existence of diagonal elements and CS. Accordingly, the discussion is divided into two cases. We begin with the simpler case:

Case 1: $h_{11}=h_{22}=h_{33}=0$.

In this case, the Hamiltonian H(k) in Equation (34) is a Hermitian operator without diagonal elements.

Indeed, we find that the Hamiltonian H(k) only possesses CS when any $h_{12},h_{13},h_{23}$ is purely imaginary. To simplify, we set $h_{1}=h_{23}i$, $h_{2}=-h_{13}i$, and $h_{3}=h_{12}i$, where $h_{1,2,3}\in R$ and *i* denotes the imaginary unit.

Therefore, the Hamiltonian H(k) in Equation (34) is rewritten as follows:

(36) $$\begin{array}{c} \begin{array}{c} H\left(k\right)=\left[\begin{array}{ccc} 0 & -h_{3}i & h_{2}i\\ h_{3}i & 0 & -h_{1}i\\ -h_{2}i & h_{1}i & 0 \end{array}\right]=h_{1}S_{x}+h_{2}S_{y}+h_{3}S_{z}, \end{array} \end{array}$$

satisfying $h_{1}^{2}+h_{2}^{2}+h_{3}^{2}=E_{2}^{2}$, and

(37) $$S_{x}=\left[\begin{array}{ccc} 0 & 0 & 0\\ 0 & 0 & -i\\ 0 & i & 0 \end{array}\right],S_{y}=\left[\begin{array}{ccc} 0 & 0 & i\\ 0 & 0 & 0\\ -i & 0 & 0 \end{array}\right],S_{z}=\left[\begin{array}{ccc} 0 & -i & 0\\ i & 0 & 0\\ 0 & 0 & 0 \end{array}\right].$$

are the spin-1 generators of the SU(3) group with eight Gell–Mann matrices.

As long as the condition $h_{1}^{2}+h_{2}^{2}+h_{3}^{2}=E_{2}^{2}$ is satisfied, we can assign any real number relevant with *k* to $h_{1,2,3}$.

Given the lower quasienergy band, $E_{2}\leq 0,E_{2}\in R$, such as $E_{2}=-arc\cos\left(-2a-b\cos k\right)$ in Equation (12), the corresponding non-normalized eigenstate $|\psi_{2}\rangle$ can be computed as follows:

(38) $$\begin{array}{c} |\psi_{2}\rangle =\left[\begin{array}{c} \frac{-h_{1}h_{3}-h_{2}E_{2}i}{h_{1}^{2}+h_{2}^{2}}\\ \frac{-h_{2}h_{3}+h_{1}E_{2}i}{h_{1}^{2}+h_{2}^{2}}\\ 1 \end{array}\right]. \end{array}$$

Multiplying $\frac{\sqrt{h_{1}^{2}+h_{2}^{2}}}{E_{2}}$ in Equation (38) yields the following:

(39) $$\begin{array}{c} |\psi_{2}\rangle =\left[\begin{array}{c} \frac{-h_{1}h_{3}}{E_{2}\sqrt{h_{1}^{2}+h_{2}^{2}}}-\frac{h_{2}i}{\sqrt{h_{1}^{2}+h_{2}^{2}}}\\ \frac{-h_{2}h_{3}}{E_{2}\sqrt{h_{1}^{2}+h_{2}^{2}}}+\frac{h_{1}i}{\sqrt{h_{1}^{2}+h_{2}^{2}}}\\ \frac{\sqrt{h_{1}^{2}+h_{2}^{2}}}{E_{2}} \end{array}\right]. \end{array}$$

We utilize polar coordinates with $\cos\varphi =\frac{h_{1}}{\sqrt{h_{1}^{2}+h_{2}^{2}}}$, $\cos\theta =\frac{h_{3}}{E_{2}}$, and 0≤θ≤π, 0≤φ≤2π, and plug them into Equation (39) to obtain a normalized $|\psi_{2}\rangle$:

(40) $$\begin{array}{c} |\psi_{2}\rangle =\frac{1}{\sqrt{2}}\left[\begin{array}{c} -\cos\theta \cos\varphi -i\sin\varphi \\ -\cos\theta \sin\varphi +i\cos\varphi \\ \sin\theta \end{array}\right]. \end{array}$$

Then, the Berry connection $\vec{A}=\left(A_{\theta },A_{\varphi }\right)$ is constituted by the following:

(41) Aθ=iψψ∂ψ∂θ∂ψ∂θ,Aφ=iψψ∂ψ∂φ∂ψ∂φ.

Then, we substitute it into $|\psi_{2}\rangle$ in Equation (40) to obtain the following:

(42) $$\begin{array}{c} A_{\theta }=0,A_{\varphi }=-\cos\theta . \end{array}$$

Here, the Berry curvature $\vec{F}=\nabla \times \vec{A}$ reads as follows:

(43) $$\begin{array}{c} F_{\theta \varphi }=\frac{\partial A_{\theta }}{\partial \varphi }-\frac{\partial A_{\varphi }}{\partial \theta }. \end{array}$$

Subsequently, we can calculate the Chern number defined as the integral of the Berry curvature over the whole sphere as follows:

(44) $$\begin{array}{c} c=\frac{1}{2\pi }\oint_{S}d\vec{k}\cdot \vec{F}. \end{array}$$

However, we find that $|\psi_{2}\rangle$ in Equation (39) is rational, except when $E_{1}=E_{2}=E_{3}=0$. The three-fold degenerate points constitute singularities that form a complex connected region in the parameter space. For the contour integral restricted by a complex connected region, Equation (44) can be mapped to a small closed loop enclosing three-fold degenerate points. Consequently, the topological phase of quasienergy $E_{2}$ is provided by the Chern number around the three-fold degenerate points:

(45) $$\begin{array}{c} \begin{array}{c} c_{2}=\frac{1}{2\pi }\int_{0}^{2\pi }\int_{0}^{\pi }\left(\frac{\partial A_{\theta }}{\partial \varphi }-\frac{\partial A_{\varphi }}{\partial \theta }\right)d\theta d\varphi \\ \;\;\;\;\;\;\;\;\;\;\;\;=\int_{0}^{\pi }-\sin\theta d\theta \\ \;\;\;\;\;\;\;\;\;\;\;\;=-2. \end{array} \end{array}$$

For the higher energy band corresponding of $E_{3}=-E_{2}$, the chiral symmetry (CS) of the Hamiltonian H(k) guarantees that the Chern number $c_{3}=2$, as calculated through the same analytical method.

As far as the flat band $E_{1}=0$, its eigenstate takes the following polar coordinates form:

(46) $$\begin{array}{c} |\psi_{1}\rangle =\left[\begin{array}{c} \sin\theta \cos\varphi \\ \sin\theta \sin\varphi \\ \cos\theta \end{array}\right], \end{array}$$

so we can calculate $c_{1}=0$.

Having examined the case with zero diagonal elements, we now turn to investigate whether physically meaningful solutions exist when the Hamiltonian contains nonzero diagonal elements. This constitutes Case 2 of our analysis, which we present below.

Case 2: $h_{11}\neq 0,h_{11}\in R$.

For Hermitian Hamiltonians with nonzero real diagonal elements, we find that this Hamiltonian H(k) does not possess CS, and the Chern number is meaningless.

Our analysis demonstrates that for Hermitian Hamiltonians with vanishing diagonal elements that satisfy both CS and the quasienergy conditions specified in Equation (35), the topological phases of both bulk spectra and edge states are characterized by Chern numbers $\left|c_{2,3}\right|=2$ in Figure 3 and Figure 4. In particular, there is a maximum peak velocity when k=0 in the dispersion relations of bulk spectra, exhibiting that three-fold degenerate points stand for the spread. Hence, we conclude that the nontrivial topological phases of bulk spectra suggest the ballistic spread for the generalized 3QWs and Szegedy’s quantum search.

Varied with dispersion relations, gapless quasienergies with $\left|c_{2,3}\right|=2$ can transform into gapped c=0. This demonstrates that Szegedy’s quantum walk provides a versatile platform for simulating such topological transitions.

In short, the results in this section indicate that Hermitian Hamiltonians H(k) possessing CS, and quasienergy conditions without diagonal elements support nontrivial topological phases. The topological phases originate from the three-fold degenerate points and are protected by chiral symmetry. In particular, nontrivial topological phases of edge states can enhance transport on marked vertices, as described in Figure 3c,d. Nontrivial topological phases of bulk spectra exhibit that Szegedy’s QW spread ballistically, explaining why three-fold degenerate points of bulk spectra and edge states are different. Compared to ill-defined topological transition points, we should point out that three-fold degenerate points have topological stability.

## 6. Conclusions

In this work, we have systematically investigated the topological phases of Szegedy’s quantum search on the 1D cycle with self-loops. We have illustrated the quasienergy dispersion relations of Szegedy’s quantum search. We have calculated topological phases based on the Chern number. We have proven that zero-diagonal Hermitian Hamiltonians satisfying both chiral symmetry and our quasienergies conditions have nontrivial topological phases. We have found that topological phases originating from three-fold degenerate points are protected by chiral symmetry. Owing to nontrivial edge states, quantum search enhances transport on the marked vertices, and the nontrivial bulk spectra are responsible for ballistic spread for Szegedy’s QW on the 1D cycle with self-loops, although the presence of self-loops seems to slow down the QWs. This result is in agreement with the generalized 3QWs [32]. What is more, in analogy with split-step QWs, Szegedy’s QW can be engineered to simulate topological phase transitions.

In contrast to Hermitian operators, systems with gain and loss or open boundaries are expressed by non-Hermitian Hamiltonians. More surprisingly, non-Hermitian systems exhibit unique topological properties absent in Hermitian counterparts, such as complex spectra [18], half integers [33,34], and non-Hermitian skin effects responsible for the bulk-boundary correspondence [35,36,37]. While non-Hermitian topological systems remain less understood than Hermitian cases, they present compelling opportunities for future research.

## Data Availability

Data are contained within the article.
